# Calculus Removed by Dilatation of the Urethra

**Published:** 1851-04

**Authors:** R. Davey


*Calculus Removed by Dilatation of the Urethra.* By R. DAVEY.—Georgiana Bingham, a little girl, aged about three years, was brought to my house, apparently in the last stage of atrophy; she had been under medical treatment for some time without any benefit; she suffered from a prolapsed state of the rectum, constant desire to make water, could scarcely get any sleep, and the right eye completely turned from straining. Suspecting stone, I sounded, and found one of considerable size; proceeded, with the assistance of my son, to remove it by dilatation; commenced with a warm hip bath, then with several bougies, and, having dilated the urethra sufficiently to introduce one of Fergusson's three-bladed dilators, grasped the stone with the forceps; but, unfortunately, it broke, therefore only a portion was brought away; the child was now very much exhausted from crying, so had her placed in bed, after I had injected the bladder with warm water. She went on well for six days, when stoppage again came on; proceeded as before, and this time brought away a much larger piece. From this period she went on well; and at the end of a fortnight gave her some preparation of iron. The child is now perfectly restored, and is getting fat, the bladder having regained its functions, and she full command of the urine. Weight of the stone removed 6 drs. 20 grs.—*London Medical Times.*

